# Exome Sequence Data of Eight SLC Transporters Reveal That *SLC22A1* and *SLC22A3* Variants Alter Metformin Pharmacokinetics and Glycemic Control

**DOI:** 10.3390/ph17101385

**Published:** 2024-10-17

**Authors:** Monserrat I. Morales-Rivera, Radamés Alemón-Medina, Angélica Martínez-Hernández, Cecilia Contreras-Cubas, Nelly F. Altamirano-Bustamante, Josefina Gómez-Garduño, Elvia C. Mendoza-Caamal, J. Orlando Nuñez-González, Raquel García-Álvarez, Cristina Revilla-Monsalve, José Antonio Valcarcel-Gamiño, José Rafael Villafan-Bernal, Federico Centeno-Cruz, Humberto García-Ortiz, Francisco Barajas-Olmos, Lorena Orozco

**Affiliations:** 1 Immunogenomics and Metabolic Diseases Laboratory, Instituto Nacional de Medicina Genómica, SS, Mexico City 14610, Mexico; mrivera@inmegen.edu.mx (M.I.M.-R.); amartinez@inmegen.gob.mx (A.M.-H.); ccontreras@inmegen.gob.mx (C.C.-C.); emendoza@inmegen.gob.mx (E.C.M.-C.); valcarceldata@gmail.com (J.A.V.-G.); joravibe@hotmail.com (J.R.V.-B.); fcenteno@inmegen.gob.mx (F.C.-C.); hgarcia@inmegen.gob.mx (H.G.-O.); 2 Postdoctoral Researcher, Consejo Nacional de Humanidades Ciencias y Tecnologías, Mexico City 14610, Mexico; 3 Pharmacology Laboratory, Instituto Nacional de Pediatría, SSA, Mexico City 04530, Mexico; ranapez@hotmail.com (R.A.-M.); josefinagomgar@yahoo.com.mx (J.G.-G.); alvarez_gar@hotmail.com (R.G.-Á.); 4 Endocrinology Service, Instituto Nacional de Pediatría, SSA, Mexico City 04530, Mexico; glutation2020@gmail.com; 5 Medical Research Unit in Metabolic Diseases, UMAE Hospital de Cardiología, Centro Médico Nacional Siglo XXI, IMSS, Mexico City 06720, Mexico; cristina_revilla@hotmail.com

**Keywords:** metformin response, pharmacokinetics, pharmacogenetics, SLC transporters, type 2 diabetes, SNVs

## Abstract

**Background**: Type 2 diabetes (T2D) is one of the leading causes of mortality and is a public health challenge worldwide. Metformin is the first-choice treatment for T2D; its pharmacokinetics (PK) is facilitated by members of the solute carrier (SLC) superfamily of transporters, it is not metabolized, and it is excreted by the kidney. Although interindividual variability in metformin pharmacokinetics is documented in the Mexican population, its pharmacogenomics is still underexplored. We aimed to identify variants in metformin SLC transporter genes associated with metformin PK and response in Mexican patients. **Methods**: Using exome data from 2217 Mexican adults, we identified 86 biallelic SNVs in the eight known genes encoding SLC transporters, with a minor allele frequency ≥ 1%, which were analyzed in an inadequate glycemic control (IGC) association study in T2D metformin treated patients. Metformin PK was evaluated in a pediatric cohort and the effect of associated SNVs was correlated. **Results**: Functional annotation classified two SNVs as pathogenic. The association study revealed two blocks associated with IGC. These haplotypes comprise rs622591, rs4646272, rs4646273, and rs4646276 in *SLC22A1*; and rs1810126 and rs668871 in *SLC22A3*. PK profiles revealed that homozygotes of the *SLC22A1* haplotype reached lower plasma metformin concentrations 2 h post administration than the other groups. **Conclusions**: Our findings highlight the potential of pharmacogenomics studies to enhance precision medicine, which may involve dosage adjustments or the exploration of alternative therapeutic options. These hold significant implications for public health, particularly in populations with a high susceptibility to develop metabolic diseases, such as Latin Americans.

## 1. Introduction

Type 2 diabetes (T2D) is a chronic disease and a worldwide public health problem. According to the Pan American Health Organization (PAHO) and World Health Organization (WHO) in the Americas, diabetes is among the six principal causes of morbidity and mortality [1] and is the leading cause of blindness, kidney failure, heart attack, stroke, and lower limb amputation [2], so emphasizing the critical importance of adequate glycemic control, not only in T2D but also in prediabetic states, is crucial for their prevention.

Metformin has emerged as the first choice for T2D treatment and is useful in managing obesity, polycystic ovarian syndrome, gestational diabetes, and cancer prevention [3,4,5]. Its broad therapeutic applications are attributed to its pleiotropic activity: it inhibits gluconeogenesis and reduces fatty acid synthesis, improves insulin sensitivity [6], and even modulates the composition of the microbiome [7,8]. Metformin exerts its effects by decreasing endogenous glucose production through the inhibition of mitochondrial respiratory chain complex I and glycerophosphate dehydrogenase in hepatocyte mitochondria [9].

Oral metformin is absorbed by enterocytes and travels through the portal vein to the liver [10]. Once metformin has exerted its action at hepatocytes, a proportion of the drug is excreted in feces, but the majority reaches the systemic blood and is eliminated by the kidneys [10,11,12]. Metformin’s passage through these tissues is mediated by members of the solute carrier (SLC) superfamily proteins. Plasma monoamine transporter (PMAT, encoded by *SLC29A4*) and organic cation transporter 3 (OCT3, *SLC22A3*) are primarily responsible for metformin’s enterocyte entry [10,11,12]. Drug entrance to the liver is mediated by the organic cation transporter 1 (OCT1, *SLC22A1*), which, along with OCT3, facilitates its passage to the systemic circulation. Organic cation transporter 2 (OCT2, *SLC22A2*) allows renal uptake, while multidrug and toxin extrusion 1 and 2 proteins (MATE1 and MATE2-K, encoded by *SLC47A1* and *SLC47A2*, respectively) are responsible for metformin’s urinary elimination [10,11,12]. Additionally, some studies have provided insights into other SLC members, such as carnitine/organic cation transporter (OCTN1, *SLC22A4*) [13] and thiamine transporter 2 (THTR2, *SLC19A3*) [14], which could participate in the transport of metformin; however, their active participation on metformin pharmacokinetics (PK) has been scarcely documented.

Because not all individuals benefit from metformin, several investigations have focused on interindividual variability in metformin response. Two independent studies found that 43% of T2D adults [15] and more than 50% of T2D children displayed metformin failure [16]. It has been noted that these observations could be associated with genetic factors, particularly single nucleotide variants (SNVs) in genes encoding SLC transporters involved in metformin uptake. One of the most studied SNVs is the nonsynonymous OCT1 change p.Arg61Cys (rs12208357), which along with other variants, has been associated with increased blood glucose concentration [17], higher renal clearance in healthy Caucasian individuals [18], and increased metformin area under the curve between 0 and 48 h (AUC_0–48h_) in healthy admixed Brazilians [19]. A computational analysis predicted that this variant, located in the extracellular domain of the protein, decreases OCT1 affinity for the metformin [20]. On the other hand, different studies on rs316019 in *SLC22A2* (OCT2), a broadly analyzed SNV, have reported controversial results about its association with metformin response [21,22,23,24]. It has been associated with altered drug elimination [25], and is predicted as possibly damaging, according to in silico analysis [20,26].

MATE1, an extrusion well-studied transporter in PK research, has shown that the intronic SNV rs2289669 in this gene is associated with reduced HbA1c levels [27,28,29,30]. Conversely, variant rs8065082 has been associated with a poor metformin response [23]. Moreover, rs2252281, an SNV located in the *SLC47A1* (MATE1) promoter region, has been associated with a decreased glucose curve in healthy adults administered with metformin [31]. We recently identified the p.Leu125Phe MATE1 variant (c.373C>T, *SLC47A1*) associated with the accumulation of metformin and lactate in the plasma [32]. Notably, in Mexican Mestizo individuals, this variant has a frequency of 16%, while in Amerindians its frequency increases to 27%; however, in other populations, it is rare or absent [32].

Despite the increasing body of research on SNVs associated with metformin PK or inadequate glycemic control (IGC), studies involving Amerindian-origin populations remain limited [33]. Here, we analyzed exome sequence data to assess the frequency of SNVs across eight metformin SLC transporter genes in a sample comprising 2217 Mexican individuals. We identified genetic association with IGC in a subset of 375 T2D patients undergoing metformin treatment. Additionally, we investigated the association between genotype and PK in an independent sample of 23 children, providing insights into the genetic factors involved in metformin response in our population.

## 2. Results

### 2.1. Variants on SLC Superfamily Metformin Transporters

**Figure 1:**
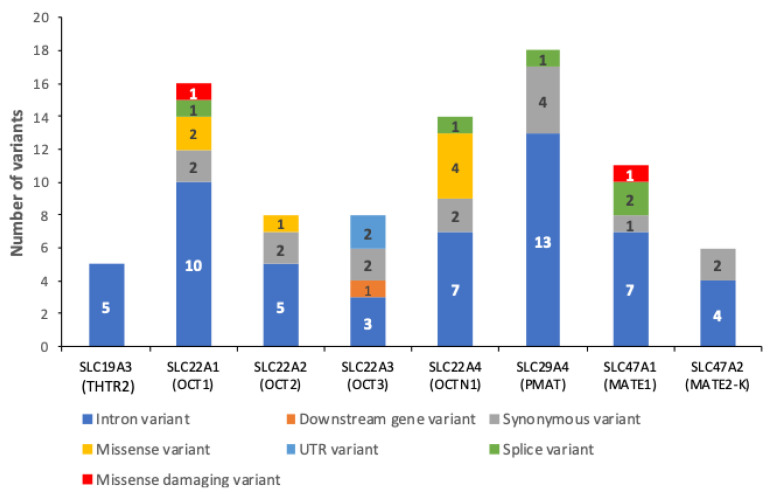
In silico functional annotation of the 86 SNVs identified in eight metformin transporter genes in the discovery sample, using the Variant Effect Predictor (VEP) software. Each bar shows the VEP consequences classification of the analyzed variants in SLC transporters, the number of genetics variants of each classification is shown within the bar. The red color shows two SNVs classified as pathogenic in *SLC22A1* (rs2282143) and *SLC47A1* (rs77474263), respectively.

In order to identify all variants on SLC superfamily metformin transporter genes *SLC22A1* (HGNC:10963, OCT1), *SLC22A2* (HGNC:10966, OCT2), *SLC22A3* (HGNC:10967, OCT3), *SLC22A4* (HGNC:10968, OCTN1), *SLC29A4* (HGNC:23097, PMAT), *SLC47A1* (HGNC:25588, MATE1), *SLC47A2* (HGNC:26439, MATE2-K), and *SLC19A3* (HGNC:16266, THTR2), we analyzed the exomes of 2217 Mexican adults from the DMS and MAIS cohorts (see the Section 4) [34,35]. This analysis revealed 86 SNVs with a frequency of at least 1% (Figure 1, Table S1); most of these variants have not been previously associated with metformin response, pharmacokinetics, impaired transporter function, or adverse effects in other populations or in vitro assays. Of these, *SLC29A4* had a high number of variants (*n* = 18) while *SLC19A3* had only five intronic variants. The variant annotation process revealed five splice region variants (rs2304081, rs7762846, rs11979775, rs2247437, and rs2247436). According to SIFT and PolyPhen analyses, p.Leu125Phe MATE1 (rs77474263 in *SLC47A1*) and p.Pro341Leu OCT1 (rs2282143 in *SLC22A1*) are damaging missense SNVs, while p.Ala87Pro OCTN1 (rs760190076 in *SLC22A4*), p.Thr88Pro OCTN1 (rs765948801 in *SLC22A4*), p.Ile306Thr OCTN1 (rs272893 in *SLC22A4*), p.Leu503Phe OCTN1 (rs1050152 in *SLC22A4*), p.Leu160Phe OCT1 (rs683369 in *SLC22A1*), p.Met408Val OCT1 (rs628031 in *SLC22A1*), and p.Ser270Ala OCT2 (rs316019 in *SLC22A2*) are classified as benign missense variants.

### 2.2. Genetic Association with Inadequate Glycemic Control

To analyze a possible genetic association with inadequate glycemic control (IGC), we evaluated the 86 SNVs in adult patients treated with metformin (*n* = 375). A diagram of sample selection for this analysis is shown in Figure S1. We found that most of the diabetic patients were in IGC (*n* = 274, 73.1%), while the remainder were under adequate glycemic control (AGC, *n* = 101, 26.9%). The anthropometric characteristics of the cohorts are shown in Table 1.

A case-control association study was performed under an additive logistic regression model adjusted for confounders (see the Section 4). This analysis identified six SNVs significantly associated with IGC (*p* < 0.05): rs4646272/T, rs4646273/G, rs4646276/G, and rs622591/C in SLC22A1, and rs668871/C and rs1810126/T in SLC22A3 (Table 2).

To assess the independence of signals between SLC22A1 and SLC22A3, we performed a conditional analysis based on the top associated SNV of SLC22A1 (rs622591). After conditioning, no other SLC22A1 variant remained significantly associated. Meanwhile, the signal produced by rs668871/C in the SLC22A3 region remained significant (OR: 2.097, 95% CI: 1.379–3.188, *p*-value = 0.0005), confirming the independence of both signals (Figure S2).

### 2.3. Linkage Disequilibrium Analysis

**Figure 2:**
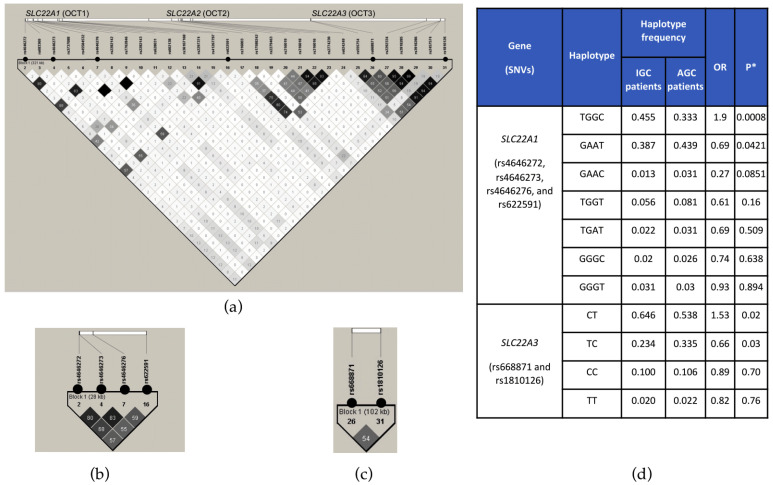
LD analysis for the region harboring the variants in chromosome 6. (**a**) Diagram of the region located between base pairs 160,551,093 and 160,872,151 on chromosome 6; it includes all SNVs within *SLC22A1*, *SLC22A2*, and *SLC22A3*. (**b**) LD analysis of SNVs associated with IGC in *SLC22A1* (rs4646272, rs4646273, rs4646276, and rs622591). (**c**) LD analysis of SNVs associated with IGC in *SLC22A3* (rs668871 and rs1810126). The grayscale range represents the pair-wise R^2^ between the SNVs, ranging from 0 (white) to 1 (black). Black circles near the SNV name indicate the associated SNVs. (**d**) IGC haplotype association analysis of blocks on *SLC22A1* and *SLC22A3*, * = *p*-value adjusted by sex, age, BMI, combined therapy, years of T2D diagnosis, and ancestry.

We conducted a linkage disequilibrium (LD) analysis focused on the region harboring associated variants in SLC22A1 and SLC22A3, covering all the variants in the region (chromosome 6:160551093-160872151) identified in this study. This analysis displayed two distinct blocks composed of the associated variants. In the first one, in SLC22A1, rs4646273 exhibited LD with rs4646276 (R^2^ = 0.83), with rs4646272 (R^2^ = 0.80), and with rs622591 (R^2^ = 0.55). Interestingly, this block was located within the gene enhancer region of the gene (Figure S3). The second block was located in SLC22A3, where rs668871 and rs1810126 were in LD (R^2^ = 0.54) (Figure 2a–c). The association analysis showed that TGGC and CT haplotypes, in SLC22A1 and SLC22A3, respectively, were significantly associated with IGC (OR = 1.9, *p* = 0.0008, and OR = 1.53, *p* = 0.02, respectively) (Figure 2d), while their counterpart haplotypes displayed a protective effect (SLC22A1/GAAT, OR = 0.69, *p* = 0.0421, and SLC22A1/TC, OR = 0.66, *p* = 0.03). Notably, although these variants are distributed worldwide, the LD analysis revealed population-specific haplotypes in the Native American-derived populations compared with other 1000 Genomes Project populations (Figure S4).

### 2.4. The slc22a1 Haplotype Associated with IGC Alters Metformin Pharmacokinetics

**Figure 3:**
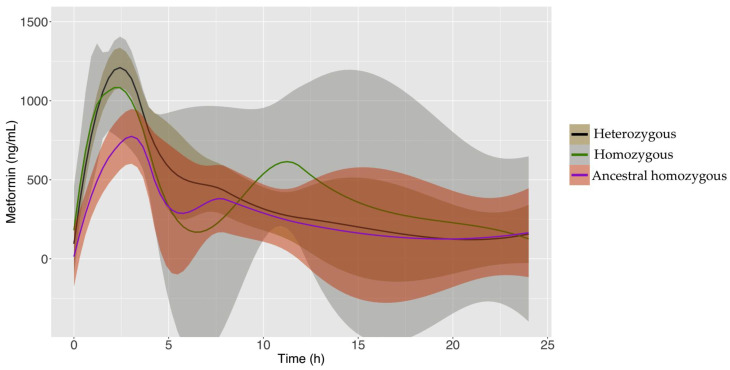
The 24-h plasma metformin concentration curve according to SLC22A1 rs4646272 and rs622591 SNVs. The ancestral homozygous haplotype carriers (purple line) reached only 45.9% of the metformin C_max_ observed in their homozygous (green line) counterparts between the second and the third hour of metformin administration.

To assess whether the top haplotype associated with IGC (TGGC) alters metformin PK, we genotyped the two SNVs that delimit the SLC22A1 block (rs4646272 and rs622591) in a sample of 23 pediatric patients undergoing metformin treatment. In parallel, a 24 h metformin concentration curve was obtained from each pediatric patient (*n* = 23). The demographic and clinical characteristics of the pediatric patients are shown in Table 1. In this population, 26.1% (*n* = 6) of patients were homozygous for the ancestral alleles (rs4646272/T and rs622591/C), 43.5% (*n* = 10) were heterozygous, and 30.4% (*n* = 7) were homozygous for protective alleles. PK analysis in the pediatric population showed that patients carrying the TC haplotype in SLC22A1 reached only 45.9% (493.4 ng/mL) of the metformin maximum concentration (C_max_) observed in patients homozygous for the protective haplotype (1074.4 ng/mL), (β = −369.0 ng/mL, P_2h_ = 0.0481) (Figure 3).

## 3. Discussion

The prevalence of metabolic traits across Latin America observed in recent years has highlighted the susceptibility of these populations to diseases such as obesity and T2D. Notably, Mexico has one of the highest obesity indexes worldwide and, according to the International Diabetes Federation, Mexico is the seventh country in the global prevalence of T2D [36]. For instance, the National Mexican Survey of Health and Nutrition (ENSANUT 2022) revealed a combined prevalence of overweight and obesity of 75.2%, alongside a T2D prevalence of 18.4% in Mexican adults [37]. It is well documented that adequate glycemic control leads to the prevention or delay of associated T2D complications [38]. Moreover, from the initial stages of the COVID-19 pandemic until June 2023, 36.7% of the people who died from COVID-19 in Mexico were individuals with diabetes [39], in line with previous reports pointing out that glycemic control can make a difference in the severity of illness and risk of death from SARS-CoV-2 infection [40]. Despite significant medical efforts, the prevalence of IGC worldwide remains high [30,41]. In this study, we found that 73.1% of patients with T2D treated with metformin had IGC (HbA1c ≥ 7%). This result aligns closely with findings from ENSANUT 2022, where IGC was present in 63.9% of T2D-diagnosed patients [37]. This observation is of concern, particularly because 86.9% of T2D-diagnosed patients in Mexico are under pharmacological treatment and 67.1% of them take oral drugs [42].

The IGC has been ascribed to intrinsic or extrinsic factors, with genetic variants in the SLC superfamily transporter genes emerging as intrinsic contributors. This observation relies on investigations that have demonstrated the relevant role of SLC transporters in metformin PK, including absorption, distribution, and elimination processes. The gene-candidate pharmacogenetic studies of metformin in Latin American patients have confirmed that glycemic control is poor in the majority of Mexican patients with T2D receiving metformin, whether alone or in combination with other diabetes medications [43,44,45]. To our knowledge, our study marks the first comprehensive analysis of SLC metformin transporters to identify their association with metformin PK and IGC in patients with T2D. Our screening of metformin transporters uncovered 86 SNVs with a minor allele frequency ≥1% in the adult patient population. We observed differences in allele frequency when comparing the discovery population (*n* = 2217 adults) to the 1000 Genomes Project database. Interestingly, three SNVs were absent worldwide, seven showed a minor allele frequency ≤ 1%, while around 50% of the variants exhibited a two-fold difference in frequency (Table S1). These results highlight the importance of including a wider range of diverse populations in metformin pharmacogenetics studies. Most of these variants lacked evidence of association with metformin response, PK, impaired transporter function, or adverse effects in other populations or in vitro assays. Notably, only a small subset of these SNVs, particularly those within *SLC22A1* (OCT1), *SLC22A2* (OCT2), and *SLC47A1* (MATE1), had been consistently reported in pharmacogenetic studies. For instance, in *SLC22A1*, the rs1867351/TT genotype has been correlated with a reduction in fasting plasma glucose in the Chinese population [46] and rs683369, a nonsynonymous variant, was associated with a diabetes incidence decrease in metformin-treated overweight patients [21]. Additionally, variants such as rs12208357 in *SLC22A1* and rs316019 in *SLC22A2* are among the most reproducibly associated with PK in the literature. In contrast with these studies, we rarely encountered them in the discovery population, and they did not show an association with metformin response, in agreement with prior results among Mexicans [43,44,47].

Meanwhile, among the variants assessed for pathogenicity prediction, only two were identified as pathogenic: p.Leu125Phe in MATE1 (*SLC47A1*), as previously reported, and p.Pro341Leu in OCT1 (*SLC22A1*). However, none of these variants showed an association with IGC in our study or in the others previously reported. This lack of association may be explained by our previously documented findings that p.Leu125Phe variant increases metformin C_max_ by altering transporter permeability, leading to hyperlactatemia [11] but, as far as we know, this SNV has not been shown to affect fasting plasma glucose levels. Additionally, p.Pro341Leu (MAF = 4.8%) changes the selectivity of the OCT1 transporter for lamivudine, a drug used against viral infections, but to a lesser extent its selectivity for metformin [48].

In the association study, we identified two haplotypes associated with IGC in metformin-treated patients (Figure 2d). The first haplotype, TGGC (rs4646272, rs4646273, rs4646276, and rs622591), resides within an enhancer region of *SLC22A1* (Supplementary Figure S2), while the second one, CT (rs668871 and rs1810126), is suggested to impact the binding site for several miRNAS (miR-1205, miR-124-3p, and miR-216b) on *SLC22A3* [49], potentially influencing gene expression and metformin PK. Although both genes are located in the same locus on chromosome 6, conditional analysis with rs622591 within *SLC22A1* exhibited an independent association signal in *SLC22A3*. Further functional studies involving tissues relevant to metformin PK, such as the liver, are needed to elucidate the functional impact of these haplotypes. It is worth noting that although some independent variants included in the associated haplotypes are highly frequent worldwide, linkage disequilibrium patterns differ across populations (Figure S4). However, similar LD patterns for analyzed haplotypes can be observed in individuals of Mexican and Peruvian ancestries included in the 1000 Genomes Project [50].

*SLC22A1* (OCT1) is highly expressed in the human liver and kidneys, whereas *SLC22A3* (OCT3) exhibits a wider tissue distribution [51]. In previous studies, rs668871/C (*SLC22A3*) has been associated with increased clearance of antiretroviral treatment in HIV patients [52] as well as with waist-to-hip ratio reduction and weight gain in data from populations of various ancestries [53]. On the other hand, it has been documented that in the Chinese population, the rs1810126 TT homozygotes have a lower risk of cardiovascular disease [54]. It is worth noting that carriers of the SNVs rs668871/C and rs1810126/T have lower *SLC22A3* expression in the liver [51]. Together, these findings suggest a potential regulatory role of these variants in the PK of metformin.

Due to the rising prevalence of obesity and diabetes, along with insulin resistance in the Mexican pediatric population [55,56], there is an increase in metformin prescriptions in this group. So, to gain insight into the functional impact of the *SLC22A1* haplotype on IGC, we evaluated the effect of the tag variants rs4646272/T and rs622591/C on metformin PK in a pediatric group. The TC haplotype homozygous carriers achieved only 45.9% of the C_max_ attained by the homozygotes of the alternative allele. It is possible that the PK findings observed in our pediatric cohort could be extended to the adult population; however, further studies are needed.

All these findings provide insights into the mechanisms underlying altered gene expression and IGC, as well as dose-dependent metformin effects [57]. This could also explain why the minor alleles of rs4646272 and rs622591, had been previously associated with IGC [58] and with cardiovascular mortality [59], respectively, in metformin users with T2D. Therefore, further studies that investigate the clearance of metformin using in vitro assays and longitudinal cohorts will be useful to delve deeper into the mechanisms.

## 4. Materials and Methods

### 4.1. Study Populations

The discovery population, in which the variants in SLC transporter genes were screened, consisted of 2217 nonrelated adults: 1143 individuals belonging to the Mestizo population from the Diabetes in Mexico Study (DMS), and 1074 Mexican Native American participants belonging to the Metabolic Analysis in an Indigenous Sample cohort (MAIS), published previously as part of the Slim Initiative in Genomic Medicine for the Americas (SIGMA) Type 2 Diabetes Consortium [34,35,60]. From them, 375 patients with T2D who were undergoing treatment with metformin, either as monotherapy or combined with other T2D medications, were selected for the association study. Adult patients with T2D receiving metformin were stratified by inadequate glycemic control (IGC) and adequate glycemic control (AGC), based on their glycated hemoglobin percentage (IGC, HbA1c ≥ 7% and AGC, HbA1c < 7%), using the criteria of Mexican regulation [61] and American Diabetes Association [62].

The effects of genetic variants on metformin PK were assessed in a sample of 23 children (aged < 18 years old) recruited by the Endocrinology Service from the National Institute for Pediatrics (INP), Mexican Ministry of Health, and diagnosed with obesity or T2D. Demographic, anthropometric, biochemical, and clinical data were collected for both cohorts at the sampling site.

### 4.2. Genotypes

In the adult patient sample (*n* = 2217), SNV genotypes were obtained from previously sequenced exome data [33,34]. From those, we extracted all the variants with a minor allele frequency (MAF) ≥ 1% in the eight known genes encoding metformin SLC transporters [9,10,11,12,13]: *SLC22A1* (chr6: 160,542,846–160,579,750, OCT1), *SLC22A2* (chr6: 160,637,786–160,679,853, OCT2), *SLC22A3* (chr6: 160,769,409–160873609, OCT3), *SLC22A4* (chr5: 131,630,086–131,679,883, OCTN1), *SLC29A4* (chr7: 5,322,573–5,346,493, PMAT), *SLC47A1* (chr 17: 19,437,166–19,482,347, MATE1), *SLC47A2* (chr17: 19,581,604–19,619,897, MATE2-K), and *SLC19A3* (chr2: 228,548,478–228,571,372, THTR2), mapped in the Genome Reference Consortium Human genome build Hg37. The functional implications of the variants were documented with the Variant Effect Predictor (VEP) tool [63]. Variants were considered damaging if they were classified as deleterious by SIFT and as probably damaging by PolyPhen. In the pediatric group, parental consent was obtained for child participation and the children provided assent. Blood samples (3 mL) were drawn via the vacutainer system (BD Inc., Plymouth, UK). DNA from children’s blood samples was obtained using a Gentra Puregene Blood Kit (QIAgen Inc. Hilden, Germany) following the manufacturer’s instructions. DNA concentrations and ratios (260/230 and 280/230) were assessed using spectrophotometry with a Nanodrop instrument (Thermo, Fisher Scientific Inc., Waltham, MA, USA), and integrity was visualized in 1% agarose gel. Only samples reaching quality control criteria were genotyped. The SNVs rs622591 and rs4646272 within SLC22A1 (OCT1) were genotyped in pediatric patients by allelic discrimination with a TaqMan SNP genotyping assay on a QuantStudio 3 Real-Time PCR System AB (Thermo, Fisher Scientific, Waltham, MA, USA).

### 4.3. Pharmacokinetic Profiles in Pediatric Patients

Metformin PK in pediatric patients was determined using a previously standardized method [64]. Nine drops of blood were obtained from each patient by finger prick and collected on Whatman 903 filter paper discs at time 0, just before medication, and at 1 h, 1.5 or 2, 4, 6, 8, 12, and 24 h after. For this purpose, they went to the Endocrinology Service after fasting for 8 to 12 h and without having taken any other medication for at least 24 h prior to and during the 12 h of the study period. Each patient received a 500-mg dose of metformin (Glucophage, Roche). Metformin extraction was performed by direct precipitation with acetonitrile (ACN) and methanol. Plasma drug concentration was quantified using a previously validated analytical method based on ultra-high performance liquid chromatography (Acquity; Waters, Milford, MA, USA) and mass spectrometry. The analytical method was validated according to the criteria of the official Mexican national guideline [65], which aligns with the international criteria. The isocratic mobile phase consisted of 5 mM ammonium acetate and ACN (80:20 vol:vol), at 0.25 mL/min. For the calibration curve, an initial solution of standard metformin hydrochloride was prepared at a concentration of 1 mg/mL in deionized water, from which subsequent dilutions were made to 20, 40, 60, 80, 100, and 200 ng/mL. Calibration control points were set at 30, 70, and 150 ng/mL of metformin, representing three intermediate points of the standard curve used to evaluate the method’s precision and accuracy. Ranitidine (1 mg/mL) was used as the external standard for calibration. The maximum standard concentration value in plasma for the metformin calibration curve was set at 1800 ng/mL, which has been reported to be reached when a dose of 2550 mg of this drug is taken orally.

### 4.4. Statistical Analysis

In the adult cohort, the association between genetic variants and IGC was evaluated using an additive logistic regression model, adjusted for confounding variables such as sex, age, body mass index, metformin combined therapy, years with T2D diagnosis, and ancestry. In the pediatric cohort, the association between genetic variants and metformin concentrations was analyzed using an additive linear regression model, adjusted for sex, age, and overweight condition. In both cohorts, genotypes were coded as follows: 0 for homozygous wildtype, 1 for heterozygous, and 2 for homozygous alternative allele. To identify secondary association signals, conditional analysis was performed as previously described [66]. The LD analysis included only haplotypes with a frequency > 1% in both groups. Statistical analyses were conducted using the PLINK v.1.07 [67] and Haploview v.4.2 tools [68]. Plasma metformin concentrations are represented as means and were determined using the WinNonlin Pro v.3.1 software.

## 5. Conclusions

Even though it has been demonstrated that Amerindian communities have a very high prevalence of metabolic diseases and harbor their own risk genetic variants, Latin American populations are underrepresented in pharmacogenomic studies. Furthermore, considering the serious problem of the diabetes pandemic, its inadequate control, and the pivotal role of metformin as the first-line treatment for T2D, understanding the genetic components that influence drug response in diverse populations is crucial. Our findings shed light on the genetic determinants of suboptimal glycemic control in patients of Amerindian descent with T2D treated with metformin. By integrating exome data, metformin PK, and clinical parameters, we documented new insights into the interplay between genetic variants and metformin response. This underscores the importance of pharmacogenomic studies, particularly in populations of diverse ancestry, to improve personalized therapeutic approaches and mitigate the burden of T2D comorbidities.

## Figures and Tables

**Table 1:** Demographic and clinical data of adult and pediatric patients treated with metformin.

	Population	Adult Patients	Pediatric Patients
	*n*	375	23
	Female	71.7 (269)	36.7 (11)
%(*n*)	Male	28.3 (106)	63.3 (19)
	AGC	26.9 (101)	-
	IGC	73.1 (274)	-
	Age (years)	58 ± 12.6	13 ± 2.6
	Diagnostic time (years)	9.4 ± 8.1	-
Mean ± S.D.	BMI (Kg/m^2^)	28.3 ± 5.3	28.1 ± 4.5
	Fasting glycemia (mg/dL)	144.5 ± 70.9	126.1 ± 69.4
	HbA1c (%)	8.2 ± 2.33	6.5 ± 2.5

S.D.: standard deviation; BMI: body mass index; HbA1c: glycated hemoglobin. IGC: inadequate glycemic control; AGC: adequate glycemic control.

**Table 2:** SNVs associated with IGC in metformin-treated patients with T2D.

Gene (Transporter)	SNV	Allele Associated with IGC	Allele Frequency IGC Group	Allele Frequency AGC Group	OR	CI 95%	*p*-Value ^1^
	rs4646272	T	0.53	0.45	1.56	1.09–2.23	0.016
*SLC22A1*	rs4646273	G	0.59	0.51	1.51	1.06–2.16	0.022
(OCT1)	rs4646276	G	0.57	0.49	1.63	1.13–2.32	0.008
	rs622591	C	0.49	0.39	1.66	1.17–2.39	0.006
*SLC22A3*	rs668871	C	0.75	0.64	1.51	1.04–2.18	0.028
(OCT3)	rs1810126	T	0.67	0.56	1.49	1.04–2.15	0.028

^1.^ Adjusted by sex, age, BMI, combined therapy, T2D diagnosis years, and ancestry. IGC: inadequate glycemic control; AGC: adequate glycemic control; OR: odds ratio; CI: confidence interval.

## Data Availability

The raw data supporting the conclusions of this article will be made available by the authors on request.

## Supplementary Materials

The following supporting information can be downloaded at: https://www.mdpi.com/article/10.3390/ph17101385/s1, Table S1: Characteristics of SNVs with minor allele frequency ≥ 1% in metformin transporters; Figure S1: Flow chart of patient selection; Figure S2: Locus Zoom plots for conditional analysis; Figure S3: Chromatin state of HepG2 cells; Figure S4: LD analysis in 1000 Genomes Project populations.
