# Supplementary material for: Exome Sequence Data of Eight SLC Transporters Reveal That SLC22A1 and SLC22A3 Variants Alter Metformin Pharmacokinetics and Glycemic Control
Source: Pharmaceuticals (Basel). 2024 Oct 17;17(10):1385. doi: 10.3390/ph17101385 (PMC11510168; doi:10.3390/ph17101385)
Supplement: Supplementary file 1 [file pharmaceuticals-17-01385-s001.zip › pharmaceuticals-3194381-supplementary.pdf]

Table S1. Characteristics of SNVs with minor allele frequency ≥ 1% in metformin transporters

Variant	Position GRCh37	Allele	MAF (Adults, n=2217)	MAF (1K genomes)	Conseque-nce	Gene symbol	Protein	cDNA position	Protein position	Amino acids	SIFT	PolyPhen
rs185401751	2:228552353	T	0.041	0.0028	intron	*SLC19A3*	THTR2					
rs13025803	2:228552709	T	0.0737	0.23	intron	*SLC19A3*	THTR2					
rs73997321	2:228553070	A	0.01037	0.04	intron	*SLC19A3*	THTR2					
rs62191374	2:228563347	T	0.010	0.04	intron	*SLC19A3*	THTR2					
rs6713116	2:228567098	T	0.4478	0.16	intron	*SLC19A3*	THTR2					
rs760190076	5:131630568	C	0.01	0.04	missense	*SLC22A4*	OCTN1	433	87	A/P	tolerated(0.22)	benign(0.18)
rs765948801	5:131630571	C	0.01	0.02	missense	*SLC22A4*	OCTN1	436	88	T/P	tolerated(0.15)	benign(0.163)
rs77719114	5:131647677	T	0.019	0.015	intron	*SLC22A4*	OCTN1					
rs270607	5:131649186	A	0.29	0.37	intron	*SLC22A4*	OCTN1					
rs2073838	5:131649222	A	0.012	0.12	intron	*SLC22A4*	OCTN1					
rs3761659	5:131657784	C	0.013	0.13	intron	*SLC22A4*	OCTN1					
rs272893	5:131663062	T	0.31	0.49	missense	*SLC22A4*	OCTN1	1091	306	I/T	deleterious(0.01)	benign(0.012)
rs273909	5:131667353	G	0.1	0.1	intron	*SLC22A4*	OCTN1					
rs2304081	5:131667548	A	0.01	0.13	splice	*SLC22A4*	OCTN1					
rs272879	5:131670546	C	0.31	0.48	synonymous	*SLC22A4*	OCTN1	1356	394	T		
rs11568506	5:131671460	A	0.02	0.009*	intron	*SLC22A4*	OCTN1					
rs12777	5:131671662	G	0.01	0.02	synonymous	*SLC22A4*	OCTN1	1587	471	G		
rs272878	5:131671769	T	0.3	0.36	intron	*SLC22A4*	OCTN1					
rs1050152	5:131676320	T	0.09	0.12	missense	*SLC22A4*	OCTN1	1681	503	L/F	tolerated(0.14)	benign(0.046)
rs1867351	6:160543123	C	0.45	0.28	synonymous	*SLC22A1*	OCT1	303	52	S		
**rs4646272**	6:160551093	G	0.46	0.21	intron	*SLC22A1*	OCT1					
rs683369	6:160551204	G	0.04	0.14	missense	*SLC22A1*	OCT1	627	160	L/F	tolerated(1)	benign(0.047)
**rs4646273**	6:160551336	A	0.4	0.12	intron	*SLC22A1*	OCT1					
rs3737088	6:160553165	T	0.04	0.08	intron	*SLC22A1*	OCT1					
rs45584532	6:160553238	T	0.03	0.09	intron	*SLC22A1*	OCT1					
**rs4646276**	6:160557154	A	0.44	0.2	intron	*SLC22A1*	OCT1					
rs2282142	6:160557515	A	0.05	0.07	intron	*SLC22A1*	OCT1					
rs7762846	6:160557569	T	0.03	0.09	splice	*SLC22A1*	OCT1					

rs2282143 (P143L)	6:160557643	T	0.05	0.07	missense damaging	*SLC22A1*	OCT1	1169	341	P/L	deleterious(0)	Probably damaging (0.972)
rs628031	6:160560845	A	0.11	0.67	missense	*SLC22A1*	OCT1	1369	408	M/V	tolerated(1)	benign(0)
rs662138	6:160564476	G	0.32	0.09	intron	*SLC22A1*	OCT1					
rs36107168	6:160575979	T	0.04	0.05	intron	*SLC22A1*	OCT1					
rs2297374	6:160575985	T	0.13	0.4	intron	*SLC22A1*	OCT1					
rs41267797	6:160577011	A	0.03	0.22	synonymous	*SLC22A1*	OCT1	1650	501	V		
**rs622591**	6:160579527	C	0.49	0.65	intron	*SLC22A1*	OCT1					
rs316003	6:160645832	C	0.05	0.31	synonymous	*SLC22A2*	OCT2	1765	502	V		
rs17588242	6:160663230	C	0.48	0.41	intron	*SLC22A2*	OCT2					
rs2279463	6:160668389	G	0.18	0.12	intron	*SLC22A2*	OCT2					
rs316019	6:160670282	A	0.03	0.14	missense	*SLC22A2*	OCT2	1067	270	S/A	deleterious(0)	benign(0.37)
rs316018	6:160670494	A	0.05	0.29	intron	*SLC22A2*	OCT2					
rs316016	6:160671500	T	0.05	0.29	intron	*SLC22A2*	OCT2					
rs2774230	6:160677614	G	0.04	0.27	intron	*SLC22A2*	OCT2					
rs624249	6:160679400	A	0.07	0.26	synonymous	*SLC22A2*	OCT2	649	130	T		
rs555754	6:160769423	A	0.28	0.42	5 prime UTR	*SLC22A3*	OCT3	124				
**rs668871**	6:160769811	T	0.29	0.42	synonymous	*SLC22A3*	OCT3	512	120	R		
rs2292334	6:160858188	G	0.34	0.7	synonymous	*SLC22A3*	OCT3	1385	411	A		
rs3918285	6:160868654	C	0.34	0.72	intron	*SLC22A3*	OCT3					
rs3918286	6:160868668	A	0.34	0.72	intron	*SLC22A3*	OCT3					
rs2457574	6:160868701	A	0.09	0.5	intron	*SLC22A3*	OCT3					
**rs1810126**	6:160872151	C	0.34	0.71	3 prime UTR	*SLC22A3*	OCT3	1886				
rs182851624	6:160874604	A	0.01	0*	downstream gene	*SLC22A3*	OCT3					
rs12540125	7:5327680	A	0.39	0.3	intron	*SLC29A4*	PMAT					
rs6952755	7:5334289	C	0.46	0.71	intron	*SLC29A4*	PMAT					
rs6948544	7:5334321	G	0.49	0.71	intron	*SLC29A4*	PMAT					
rs4724524	7:5334469	G	0.5	0.29	intron	*SLC29A4*	PMAT					
rs59119551	7:5334582	T	0.36	0.33	intron	*SLC29A4*	PMAT					
rs11760864	7:5334636	T	0.04	0.08	intron	*SLC29A4*	PMAT					
rs181930968	7:5336526	T	0.04	0.01	intron	*SLC29A4*	PMAT					

rs13244318	7:5336885	T	0.48	0.49	intron	*SLC29A4*	PMAT					
rs62441137	7:5336903	T	0.02	0.04	intron	*SLC29A4*	PMAT					
rs11761173	7:5338693	T	0.02	0.02	synonymous	*SLC29A4*	PMAT	1118	319	G		
rs6950111	7:5338714	C	0.17	0.28	synonymous	*SLC29A4*	PMAT	1139	326	D		
rs6946155	7:5338770	G	0.13	0.16	intron	*SLC29A4*	PMAT					
rs183051177	7:5339167	T	0.03	0*	intron	*SLC29A4*	PMAT					
rs34551990	7:5340002	G	0.13	0.17	intron	*SLC29A4*	PMAT					
rs553825189	7:5340109	T	0.01	0	synonymous	*SLC29A4*	PMAT	1427	422	C		
rs3892745	7:5342388	T	0.03	0.07	intron	*SLC29A4*	PMAT					
rs11979775	7:5342413	C	0.1	0.26	splice	*SLC29A4*	PMAT					
rs149588788	7:5342474	T	0.01	0.01	synonymous	*SLC29A4*	PMAT	1658	499	S		
rs77474263 (L125F)	17:19451364	T	0.21	0.002	missense damaging	*SLC47A1*	MATE-1	459	125	L/F	deleterious(0)	Probably damaging (0.999)
rs17683662	17:19453103	G	0.06	0.005	intron	*SLC47A1*	MATE-1					
rs2247518	17:19454575	C	0.22	0.26	intron	*SLC47A1*	MATE-1					
rs368661527	17:19454603	C	0.09	0.02	intron	*SLC47A1*	MATE-1					
rs2247437	17:19454725	C	0.16	0.2	splice	*SLC47A1*	MATE-1					
rs2247436	17:19454733	A	0.24	0.22	splice	*SLC47A1*	MATE-1					
rs2440149	17:19458777	G	0.34	0.28	intron	*SLC47A1*	MATE-1					
rs16960203	17:19458972	T	0.14	0.02	synonymous	*SLC47A1*	MATE-1	794	236	L		
rs1054715	17:19460972	A	0.11	0.25	intron	*SLC47A1*	MATE-1					
rs73981546	17:19461020	T	0.15	0.04	intron	*SLC47A1*	MATE-1					
rs2289667	17:19470639	G	0.15	0.02	intron	*SLC47A1*	MATE-1					
rs4925042	17:19608773	G	0.43	0.57	synonymous	*SLC47A2*	MATE2-K	1044	331	Y		
rs12942065	17:19609947	C	0.16	0.23	intron	*SLC47A2*	MATE2-K					
rs35263947	17:19610104	T	0.38	0.31	intron	*SLC47A2*	MATE2-K					
rs4924792	17:19617236	T	0.39	0.37	synonymous	*SLC47A2*	MATE2-K	396	115	G		
rs9900497	17:19617393	T	0.35	0.39	intron	*SLC47A2*	MATE2-K					
rs117483482	17:19618582	A	0.07	0.03	intron	*SLC47A2*	MATE2-K					

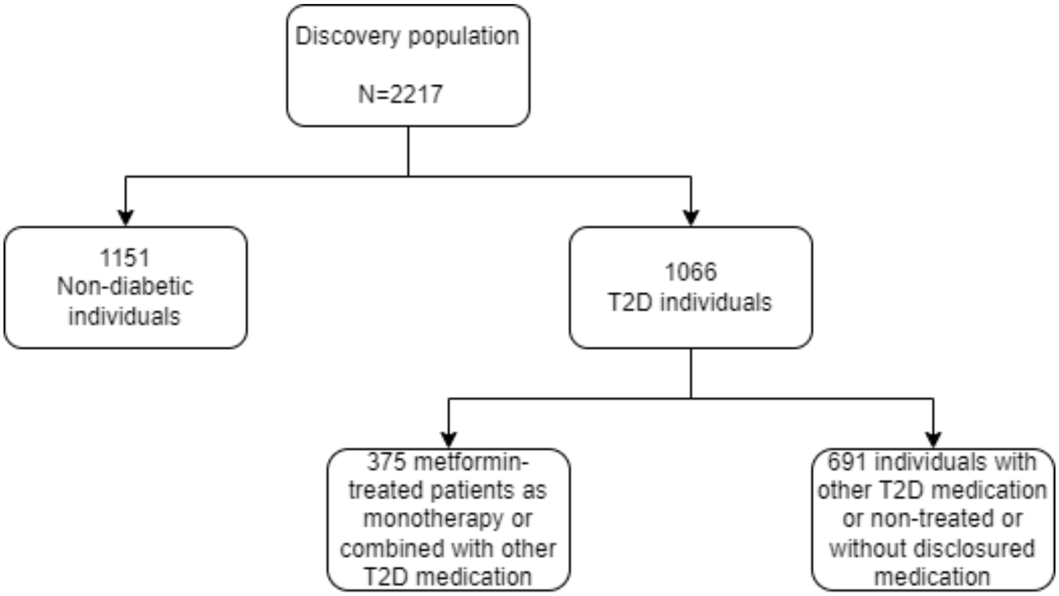

Figure S1. Flow chart of patient selection.

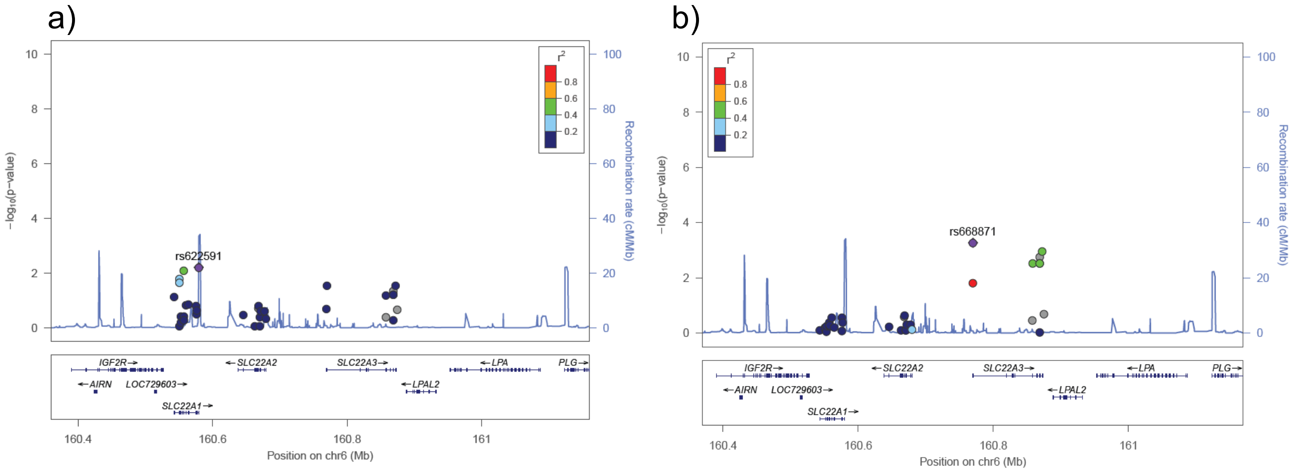

Figure S2. **Locus Zoom plots for conditional analysis**. a) Regression analysis in Locus Zoom showed rs6229591 in *SLC22A1* (OCT1) as top SNV associated with IGC in the study. b) Conditional analysis with the SNV rs622591, displayed an independent signal in the region in *OCT3* associated with IGC with rs668871 as new top SNV.

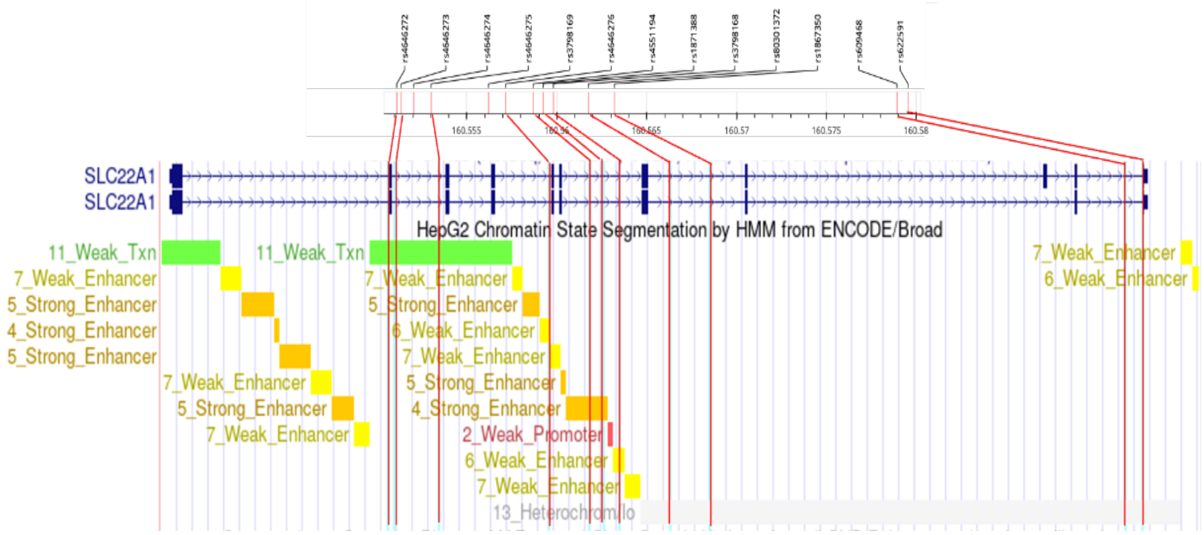

Figure S3. Chromatin state of HepG2 cells. UCSC Genome viewer of the *SLC22A1* region together with annotations to enhancer region from ENCODE. The red lines highlight the positions of SNVs conforming to the *SLC22A1* haplotype.

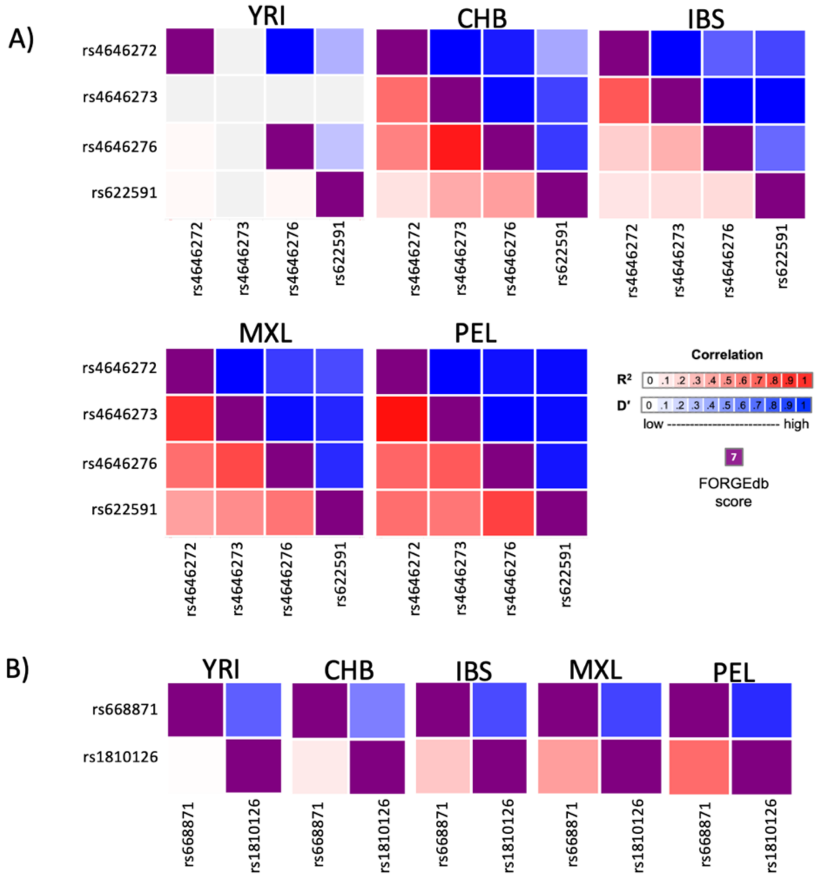

Figure S4. LD analysis in 1000 Genome Project populations. (A) LD analysis of the *SLC22A1* haplotype and (B) the *SLC22A3* haplotype in five representative populations of the 1000 Genome Project.
